# Metabolism of tumor infiltrating T cells

**DOI:** 10.3389/fimmu.2025.1711328

**Published:** 2026-01-21

**Authors:** Hildegund CJ Ertl

**Affiliations:** Wistar Institute, Philadelphia, PA, United States

**Keywords:** cancer, glycolysis, metabolism, oxidative phosphorylation, T cells

## Abstract

The microenvironment of solid tumor is commonly low in key nutrients such as glucose providing metabolic challenges for tumor infiltrating T lymphocytes (TIL), which upon activation switch to glycolysis to meet their need for energy and effector molecule production. Consequently, TIL become functionally impaired and die unless they can switch their metabolism to alternative pathways such as oxidative phosphorylation catabolizing lipids that are in ample supply within solid tumors. Medicinal interventions that alter the nutrient supply within tumors or that facilitate the TIL’s metabolic switch away from glycolysis have been tested in experimental animals and clinical trials. Some of them were shown to increase TIL functions, prolong their survival and enable them to slow tumor progression.

## 1 Introduction

All living organisms require energy to function and survive. Complex organisms acquire energy by consumption of food, which is broken down into smaller molecules, such as sugars, amino acids and fatty acids. They circulate and are taken up by cells. Within the cells they serve to generate energy and building blocks for macromolecules. Quiescent cells use minimal energy while dividing cells or cells that produce large numbers of macromolecules such as plasma cells, which can produce up to 10,000 antibody molecule per second (1), require vast amounts of nutrients and energy (2).

Mammalian cells mainly produce energy through aerobic respiration, which takes place in mitochondria and as the name implies requires oxygen (O_2_). Under anaerobic condition or under circumstances where cells require energy rapidly to allow for proliferation or enhanced production of macromolecules, they can generate adenosine triphosphate (ATP), the currency of energy, by glycolysis. This pathway that requires glucose takes place in the cytoplasm.

Even in a healthy organism cells compete for nutrients. Cells with poor fitness are replaced by those with superior metabolic capacity, a process that serves as a quality control to ensure survival and expansion of the most robust cells (3).

Within solid tumors competition for nutrients becomes detrimental to the infiltrating host cells. Cancer cells in their zest to proliferate consume large amounts of glucose and amino acids to produce building blocks for cell division and to generate energy thus depleting the TME of vital resources for TIL. Even in presence of O_2_, which due to lack of vascularization is commonly limited in some areas of solid tumors, transformed cells use mainly glycolysis, a biological phenomenon that is referred to as the Warburg effect (4–6). Activated T cells are the main immune effector cell type that can halt tumor progression or even affect regression, as has been shown by transfer of *in vitro* expanded TIL or genetically modified T cells with chimeric antigen receptors (CAR-T cells) (7–9). They also require glycolysis to become and remain fully functional (10, 11). Within the tumor microenvironment (TME) T cells and tumor cells compete for glucose, with the latter commonly outcompeting the former (12) leading to their functional impairments (13, 14). Lack of other nutrients as well as increased levels of tumor cell-derived lactate, the end product of glycolysis, within the TME further weaken the T cells’ ability to proliferate and function (15, 16).

Here we discuss the effect of metabolic constrains within the TME on TIL with emphasis on CD8^+^ T cells and potential avenues to restore their functions using melanoma as the primary example. Metabolic interventions that have thus far only been tested in other types of solid cancers are also discussed with the understanding that in this rapidly evolving field they may also benefit melanoma patients.

## 2 Main text

### 2.1 Melanoma microenvironment

Melanoma is the leading cause of skin cancer-related deaths affecting mainly light skinned individuals upon excessive exposure to ultraviolet light. Melanomas unlike many other solid tumor types are highly immunogenic; nevertheless, spontaneous remissions, that could potentially be mediated by the immune system, are exceedingly rare (17).

Melanomas are composed of transformed melanocytes and other stromal cells most notably cancer-associated fibroblasts, which support tumor growth by producing and remodeling extracellular matrix proteins, promoting angiogenesis and inhibiting immune effector functions (18). In addition, melanomas become infiltrated with immune cells such as macrophages that differentiate towards the M2 type (19). Through production of epithelial growth factor (EGF) and platelet derived growth factor (PDGF) they promote tumor cells proliferation, while their secretion of vascular endothelial growth factor (VEGF) stimulates vessel formation, which together with the release of enzymes that degrade the extracellular matrix aids tumor cell spread and formation of metastasis. M2 macrophages are immunosuppressive through production of anti-inflammatory cytokines such as interleukin (IL)-10 and transforming growth factor (TGF)-ß, which furthermore promote development and functions of regulatory T cells (Tregs). Myeloid derived suppressor cells (MDSC) are a heterogeneous population of immature myeloid cells that also inhibit immune functions, promote tumor growth and metastases formation (20). Other cells of the innate immune system that are attracted into melanomas are natural killer cells that can lyse tumor cells even if they lack expression of major histocompatibility complex (MHC) antigens and dendritic cells that are instrumental for presentation of antigen to T cells (21).

Melanoma infiltrating lymphocytes of the adaptive immune system include CD4^+^ T cells in form of T helper cells, which slow tumor progression through the release of cytokines and by promoting antigen presentation and activation of other immune effector cells (22). In contrast, CD4^+^ Tregs promote tumor cell growth by blocking immune functions (23). CD8^+^ T cells through the release of granzyme B and perforin can cause tumor cell death and are thought to be vital for immune-mediated cancer regression (24). B cells, which make up nearly one third of the infiltrating lymphocyte population in melanomas can depending on circumstances promote or suppress tumor growth (25).

### 2.2 Cell metabolism

Cell metabolism is composed of catabolism, which breaks down molecules and creates energy, and anabolism which uses energy to produce new macromolecules required for maintenance of an organisms, as building blocks for dividing cells, or as effector molecules during immune responses. Cells can generate energy through two main pathways. Glycolysis, which requires glucose and, the tricarboxylic acid (TCA) cycle, also referred to as the citric acid or Krebs cycle, which catabolizes acetyl-coenzyme (Co)A. This metabolite can be generated from carbohydrates or proteins. Alternatively, acyl-CoA derived from fatty acid catabolism can be broken down by ß-oxidation into acetyl-CoA, which can also be synthesized within the mitochondria by conversion of pyruvate, a breakdown product of glucose.

#### 2.2.1 Fatty acid catabolism

Long-chain fatty acids are taken up by cells through specific transporters while short-chain fatty acids can also enter by diffusion. Within the cytoplasm they are broken down by a ligase into acyl-CoA. Acyl-CoA derived from short-chain fatty acids can enter mitochondria directly unlike those from long-chain fatty acids. The latter have to be linked by carnitine palmitoyltransferase 1 (CPT1) to carnitine, which is spanning the mitochondrial membrane. The acyl-CoA-carnitine complexes are shuttled into mitochondria in exchange for free carnitines. Within the mitochondria acyl-CoA is released and undergoes ß-oxidation and is converted into acetyl-CoA.

#### 2.2.2 Cellular respiration

Within the mitochondria acetyl-CoA enters the TCA cycle. There through the activity of eight different enzymes it is first converted upon binding to oxaloacetate to citate then successively into cis-aconitate, d-isocitrate, a-ketoglutarate, succinyl-CoA, succinate, fumarate, malate and last, finishing the cycle, oxaloacetate. The different intermediates provide precursors for some amino acids as well as nicotinamide adenine dinucleotide (NADH) and flavin adenine dinucleotide (FADH2), which enter the electron transport chain (ETC) that is located within the inner mitochondrial membrane. Electrons from NADH or FADH2 pass through a series of reactions to O_2_ resulting in the conversion of high energy molecules to those with lower energy. This process releases energy which generates a proton gradient across the mitochondrial membrane. This allows for the phosphorylation of adenosine diphosphate (ADP) into ATP. ATP in turn releases energy when it becomes hydrolyzed into ADP and phosphate (26, 27). The Krebs cycle is a very efficient provider of energy and on average produces 30–32 ATP per molecule of glucose, the latter being the equivalent of two molecules of pyruvate.

#### 2.2.3 Glycolysis

Glycolysis is by far less efficient in producing energy than cellular respiration. It produced 4 molecules of ATP but consumes two of those resulting in a net gain of 2 ATP molecules per one molecule of glucose. With the help of 9 different enzymes glucose is converted sequentially into glucose-6-phosphate, fructose-6-phosphate, fructose 1,6-biphosphate, glutaraldehyde-3-phospharte, 1,3-biphosphoglycerate, 3-phosphoglycerate, 2-phoshoglycerate, phosphoenolpyruvate and pyruvate. Pyruvate then crosses into the mitochondria where it can be converted through pyruvate dehydrogenase into acetyl-CoA, which in turn enters the TCA cycle. Alternatively, within the cytoplasm lactate dehydrogenase can convert pyruvate to lactate, which is either secreted or, upon moving into the mitochondria, converted back to pyruvate (28). Glycolysis intermediates can enter the pentose phosphate pathway for production of nucleic acids and lipids, which are both crucial to sustain cell proliferation. They can also be used for production of amino acids and gluconeogenesis.

### 2.3 Tumor cell metabolism

Tumor cell metabolism is highly heterogenic. While some tumor cells, especially those that grow very rapidly, switch away from use of the TCA cycle and oxidative phosphorylation (OXPHOS) towards glycolysis, others continue to produce energy through cellular respiration. Alternatively, some tumor cells use both pathways. Tumor cells show metabolic flexibility and can adjust to their microenvironment (29) so that even within a solid tumor there is heterogeneity in part dictated by the supply of nutrients and O_2_. Melanoma cells primarily produce energy through glycolysis. They increase the activities of the phosphoinositide 3-kinase (PI3K) and mammalian target of rapamycin (mTOR) pathways (30), which in turn promote consumption of nutrients by enhancing cell surface expression of nutrient receptors that facilitate uptake of glucose and amino acids. The tumor cells’ switch to glycolysis is further enhanced by hypoxia inducible factor (HIF)-1α, which is upregulated by mTOR as well as by low levels of O_2_ commonly found in areas of rapidly growing solid tumors (31). HIF-1α in turn increases expression of glucose transporters such as glucose transporter (GLUT)1 (32, 33), several enzymes of the glycolytic pathway and the activity of pyruvate dehydrogenase kinase (PDK1), an enzyme that phosphorylates and inactivates the pyruvate dehydrogenase complex that is needed to convert pyruvate to acetyl-CoA (34, 35). Mutations in melanoma such as increases in copy numbers of the chromosome section that carries the Myc gene or changes in the rat sarcoma (RAS)/rapidly accelerated fibrosarcoma (RAF)/mitogen-activated protein kinase (MAPK) pathways due to mutations (36) or enhanced signaling (37) promoted by growth factors also leads to increased activity of the myc pathway, which again promotes uptake of glucose and glycolysis (38). Upon glycolysis, secretion of lactate acidifies the TME and impairs T cell functions by blocking them from lactate export leading to intracellular acidosis (13, 39).

Glutaminolysis is another metabolic pathway tumor cells use to generate factors needed for the synthesis of macromolecules (40). Consumption of glutamine is promoted by the myc and HIF-1α pathways. Other amino acids that support tumor cell growth are arginine, which is used for synthesis of amino acids and nitric oxide (41) and tryptophane (42) that is catabolized by Indoleamine 2,3-dioxygenase (IDO) to fuel the kynurenine pathway, which generates immunosuppressive metabolites such as kynurenine (43).

### 2.4 T cell metabolism

Quiescent T cells use glucose, amino acids and fatty acids to produce energy through OXPHOS (44). Ligation of the T cell receptor (TCR) to MHC/antigen derived peptide complexes with concomitant interactions between CD28 on T cells and co-stimulators such as CD80 or CD86 on antigen presenting cells initiates signaling cascades that result in reprogramming of the cells’ metabolism (45, 46). Specifically, TCR engagement promotes activation of the extracellular signal-regulated kinase (ERK)/MAPK pathways and enhances the activities of myc and HIF-1α while CD28 ligation augments PI3K/mTor/AKT signaling. This is further enforced by stimulation of the TCR and by binding of CD25 to IL-2, a key growth factor for CD8^+^ T cells, which activates PI3K upon stimulating Janus kinases (47). As a result, activated T cells switch to glycolytic energy production using similar pathways as those described above for tumor cells. Reliance on glycolysis upon activation is more pronounced in CD8^+^ than CD4^+^ T cells (48). The former, which proliferate more vigorously, have a higher glycolytic flux, while the latter upon activation only partially switch to glycolysis but also continue to generate energy through OXPHOS. This is particularly the case for Tregs, which early upon activation switch to glycolysis but then convert to energy production by fatty acid oxidization (FAO) (49). These data are based largely on *in vitro* experiments, and it should be pointed out that they are not fully supported by *in vivo* studies, which indicate that for example alloreactive effector T cells (50, 51) or T cells induced by a bacterial infection (52) not only increase energy production by glycolysis but also by mitochondrial respiration. Furthermore, Tregs are metabolically flexible and can adjust their metabolism following environmental and developmental cues (53–55).

Once antigen expressing cells have been removed, activated T cells either undergo apoptosis, or they differentiate into memory cells (56, 57). Lack of TCR and CD28 signaling causes metabolic rewiring away from energy production by glycolysis towards FAO and OXPHOS (58, 59). In comparison to naïve T cells memory T cells upon binding to IL-15, a cytokine that is essential for their maintenance and survival, develop higher mitochondrial masses with elongated mitochondria indicative of an increased spare respiratory capacity (60, 61). This results in better bioenergetic fitness, which allows them to survive and respond more vigorously upon reencounter of their antigen.

### 2.5 T cell metabolism within solid tumors

Within solid tumors T cells and tumor cells compete for glucose and other nutrients (12); the latter with their higher expression levels of glucose and amino acid receptors generally win this battle (12). Lack of key nutrients has dire consequences for activation of T cells by not only depriving them of their preferred source of energy but by also by denying them metabolites for biosynthesis of key macromolecules that are needed for cell divisions and effector functions (12–14).

As was shown *in vitro* and *in vivo*, lack of glucose impairs T cell functions by reducing production of effector molecules such as interferon-(IFN)-γ, tumor necrosis factor (TNF)-α and granzyme B (14, 62). In addition, the TME causes upregulation of so-called exhaustion markers such as programmed death (PD)-1 on T cells (63). Within hypoglycemic tumors unlike during chronic infections this is not solely driven by prolonged exposure to antigen as bystander T cells, which are specific for an unrelated antigen, also show increased expression of this exhaustion marker (64). Binding of PD-1 to programmed death-ligand (PD-L)1 or PD-L2, which are both expressed on melanoma cells, leads to phosphorylation of tyrosine residues within the immunoreceptor tyrosine-based inhibition motif (ITIM) and the immunoreceptor tyrosine-based switch motif (ITSM) located within the cytoplasmic tail of PD-1. The latter recruits Src homology 2 domain-containing phosphatase 2 (SHP-2), which dephosphorylates and thereby inhibits signaling components of the TCR, PI3K/AKT and Ras/mitogen activated protein kinase kinase (MEK)/ERK pathways (65). This inhibits proliferation and cytokine production and forces T cells to switch from glycolysis to FAO (66). Metabolism of fatty acids including their uptake, intracellular catabolism and transport of break-down products into mitochondria is controlled by peroxisome proliferator-activated receptor (PPAR)α, a nuclear receptor transcription factor. Bioenergetic stress results in stimulation of the adenosine monophosphate-activated protein kinase (AMPK) pathway, which in turn phosphorylates and activates peroxisome proliferator-activated receptor gamma coactivator (PGC)-1a (67), a transcriptional co-activator of PPARα, which increases enzymes involved in FAO and ketogenesis. The latter leads to the production of ketone bodies. Another key transcription factor that regulates fatty acid uptake, transport and oxidization is PPARδ (68).

Energy production through FAO is an alternative pathway for T cells to gain energy in a hypoglycemic TME (69). Nevertheless, FAO requires O_2_ which is commonly low in areas of rapidly growing, poorly vascularized tumors. Production of energy through ketone bodies requires less O_2_ compared to mitochondrial catabolism of glucose or fatty acids (70, 71). Glucose catabolism uses O_2_ to convert pyruvate to acetyl-CoA, to generate NADH in the citric acid cycle and as electron acceptor in the ETC. On average 1 O_2_ molecules is consumed to generate 2.6 molecules of ATP from glucose. Fatty acids require a number of oxidative steps to break down the long carbon chains into acety-CoA and for example palmitic acid consumes 1 O_2_ molecule to generate 2.3 ATP molecules. Ketone bodies require fewer steps to be converted into acetyl-CoA and have thus a more favorable ATP production to O_2_ consumption rate, which for example for acetoacetate is 3.3.

Ketone bodies have been shown to increase T cell functions such as production of cytokines and cytolysis by not only providing a source of energy but also by regulating histone acetylation at effector gene loci (72).

Depletion of the essential amino acids provides further challenges to activated T cells. Depletion of tryptophane within the TME by IDO blocks T cell proliferation (73). Lack of glutamine, another essential amino acid, that is needed for biosynthesis of purines and pyrimidines, leads to cell cycle arrest. Tumor cell-mediated depletion of glutamine in a hypoglycemic environment in addition elicits a cellular stress response in T cells (74), which activates the AMPK pathway. Stimulation of AMPK in turn inhibits energy consuming processes (75) again blocking cell proliferation (76). Lack of arginine reduces the activities of cyclin D3 and cyclin-dependent kinase (CDK)4 in T cells; both are essential for cell divisions (77). Arginine depletion by tumor cells furthermore inhibits CD8^+^ TCR expression; it decreases the amount of the CD3ζ chain by inhibiting biosynthesis of this most rapidly cycling component of the TCR (78).

Lack of nutrients within the TME results in an accumulation of ADP and AMP. This causes upregulation of AMPK in TIL (79). AMPK restores energy homeostasis by inhibiting anabolic processes such as protein or lipid synthesis and gluconeogenesis while increasing catabolic pathways such as FAO. Specifically, AMPK increases cell surface expression of fatty acid transporters. It inhibits acetyl-CoA carboxylase, which increases production of malonyl-CoA, a primary intermediate of fatty acid biosynthesis. Malonyl-CoA also is a potent inhibitor of the FAO rate-limiting enzyme Cpt1a, which moves fatty acids from the cytoplasm into the mitochondria (80).

Notwithstanding, in spite of alternative sources of energy activated T cells fare poorly in a TME. They lose functions, their numbers progressively decrease, and they are decapacitated to a point that they pose no threat to tumor progression. This is exemplified by the lack of reliable therapeutic benefits of adaptive transfer of TIL or CAR-T cells in many cancer patients (81–84); the transferred cells commonly die rapidly and although this can have multiple causes, it in part reflects their metabolic dysfunctions.

### 2.6 Medicinal interventions to improve TIL metabolism

The bioenergetic fitness of TIL and thus their ability to inhibit tumor progression can be improved by changing the nutrient supply within the TME or by rewiring the T cells’ metabolism.

#### 2.6.1 Altering the nutrient supply within the TME

**Figure 1:**
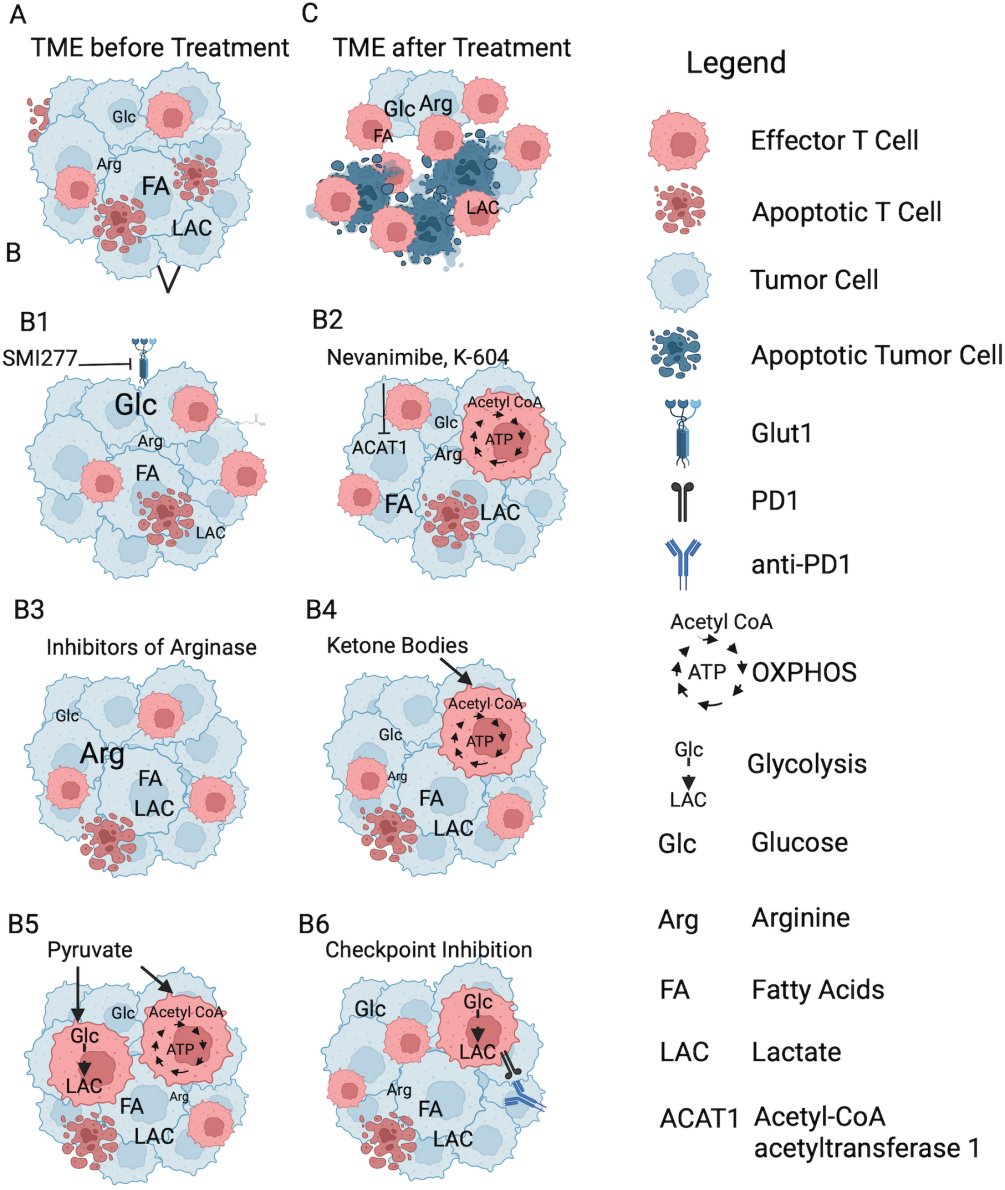
The effects of drugs that affect tumor cell metabolism on the TME. **(A)** TME before treatment. In this and the following graphs the font size of the metabolites reflects the amounts present in the TME. **(B)** Treatments: [B1] SMI2777 inhibits glucose (Glc) uptake by Glut1 which increases the Glc content in the TME. [B2] Nevanimibe and K-604 inhibit ACAT1 and increase fatty acids (FA) within the TME. [B3] Inhibitors of arginase increase levels of arginine within the TME. [B4] Ketone bodies serve as an efficient source to fuel OXPHOS in TIL. [B5] Pyruvate supplementation increases glycolysis and OXPHOS in TIL. [B6] Checkpoint blockade increases glycolysis by TIL. **(C)** Either intervention reduces tumor cell viability; they also improve T cell survival functions. Created with BioRender.com.

The nutrient supply within the TME (**Figure 1A**) can be changed by drugs, which modify tumor cell metabolism or alternatively by dietary changes (**Figures 1B, C**). Of note, such treatments have to be approached with caution as they are commonly not selective and affect other cells, such as TIL, as well (85).

##### 2.6.1.1 Metabolic drugs

###### 2.6.1.1.1 Inhibitors of glucose uptake

Inhibitors of glucose uptake such as the small molecule drug SMI277 reduce glucose uptake into tumor cells by inhibiting Glut1 causing their cell cycle arrest and apoptosis (**Figure 1B1**). Blocking the tumor cells’ ability to consume glucose results in increased levels of this nutrient within the TME while decreasing the amount of secreted lactate. In experimental animals SMI277 was shown to reduce tumor progression and improve TIL functions, which presumably reflects their superior fitness in a metabolically more favorable TME (86).

SMI277 like other Glut1 inhibitors is not selective for tumors cells and thereby can cause severe dysregulation of an individual’s metabolism such as hyperglycemia or ketoacidosis. Also, tumor cells can adapt to such inhibitors by switching to alternative glucose receptors such as Glut4. To date the efficacy of this approach has only been tested *in vitro* or in animal models (87). In addition to toxicity Glut1 inhibitors will also most likely inhibit CD8^+^ TIL activation, which may further negate potential benefits of depriving tumor cells of one of their key nutrients.

###### 2.6.1.1.2 Inhibitors of enzymes of fatty acid metabolism

In some types of cancer acyl-CoA:cholesterol acyltransferase (ACAT)1 inhibitors such as nevanimibe and K-604 have been shown to reduce tumor progression and enhance TIL functions by downregulating cholesterol synthesis and energy production in tumor cells while enhancing FAO and OXPHOS in T cells (**Figure 1B2**) (88–91). ACAT1 has multiple functions. It is involved in ketogenesis and ketolysis, it plays a role in the degradation of isoleucine and fatty acids, and it can acetylate and thereby inhibit other proteins such as pyruvate dehydrogenase. Efficacy was shown in preclinical experiments with both drugs. Nevanimibe, when tested in a clinical trial in patients with adrenocortical carcinoma lacked efficacy due to dose-limiting toxicity.

Inhibitors of another enzyme of lipid metabolism. i.e., stearoyl-CoA desaturase (SCD)-1, which converts saturated fatty acids into monounsaturated fatty acids, the substrates for ACAT1, are being explored for cancer therapy. They have been shown to improve TIL functions and to enhance the effectiveness of checkpoint blockade (92).

Pre-clinically the drugs showed significant toxicity in part due to inflammatory reactions triggered by an accumulation of saturated fatty acids (93).

###### 2.6.1.1.3 Inhibitors of enzymes of amino acid metabolism

Modulators of amino acid metabolism may also shift the balance in the battle between tumor cells and TIL. Irreversible inhibitors of glutaminase (GLS), which converts glutamine into glutamate, a substrate for the TCA cycle, reduce tumor progression but also impair TIL functions (94). The glutamase inhibitor telaglenastat (CB-839) if combined with other therapeutic interventions has shown promise in initial clinical trials with an acceptable safety profile and some reduction in tumor progression (95, 96). In a follow-up trial telaglenasta was combined with a check-point inhibitor; addition of the drug did not improve efficacy (97). Others, such as Bis-2-(5-phenylacetamido-1,2,4-thiadiazol-2-yl)ethyl sulfide (BPTES) or DRP-104 (sirpiglenastat) have only been tested preclinically or have just entered clinical trials. (ClinicalTrials.gov ID NCT04471415, 101).

The high arginase activity in many types of cancers is caused by its production by MDSCs. Arginase converts L-arginine into urea and L-ornithine leading to its depletion in the TME, which is detrimental for TIL functions. Inhibition of arginase by for example the small-molecule inhibitor OATD-02 or by CB-1158 has been shown to reduce cancer progression (98) and metastasis formation (99) and to enhance TIL functions (100, 101). In addition they were shown to blunt the immunosuppressive effects of MDSCs (102) (**Figure 1B3**) in pre-clinical experiments and they are currently being tested in phase I trials (ClinicalTrials.gov ID: (NCT05759923, NCT02903914).

The effects of inhibitors of amino acid metabolisms are not tumor cell specific resulting in off-target effects. Furthermore, the inhibitors if based on bacterial proteins can elicit antibody responses or allergic reactions precluding their repeated use. Tumor cells can become resistant by shifting to alternative metabolic pathways.

##### 2.6.1.2 Dietary therapies

###### 2.6.1.2.1 Ketone bodies

Preclinical (103) and early clinical (104) trials have shown that a ketogenic diet may slow tumor progression. Some types of tumor cells including melanoma cells, unlike T cells, cannot metabolize ketone bodies due to mitochondrial dysfunctions or lack of ketolytic enzymes such as succinyl-CoA:3-oxoacid CoA transferase (SCOT), which catalyzes the transfer of coenzyme A from succinyl-coenzyme A to acetoacetate, mitochondrial acetoacetyl-CoA thiolase, which cleaves acetoacetyl-CoA into two acetyl-CoA molecules, and D-beta-hydroxybutyrate dehydrogenase (BDH1) that converts acetoacetate to D-beta-hydroxybutyrate (105, 106).

As was shown in cell culture or in experimental animals, ketone bodies reduce expression of glycolytic enzymes (107) and lactate transporters (108), decrease uptake of key amino acids (109), and reduce the activity of myc (107). This together can inhibit tumor cell proliferation, while at the same time increasing the T cells’ nutrient supply and reducing their stress due to acidosis (**Figure 1B4**).

A ketogenic diet, which is high in fat and low in carbohydrates is in general well tolerated although in some patients it causes gastrointestinal problems, kidney stones or hyperlipidaemia. In a mouse breast cancer model, they were shown to increase metastasis formation (110). Ketone bodies may not be universally beneficial for tumor patients as some cancer cells can use them to promote their metabolism and clinical trials thus far have yielded inconclusive results (111).

###### 2.6.1.2.2 Pyruvate supplementation

TIL show reduced activity of enolase 1, a glycolytic enzyme, which converses 2-phosphoglyceric acid into phosphoenolpyruvic acid. This results in a reduction of pyruvate, which is essential to allow glucose to fuel the TCA cycle. Bypassing this enzyme by providing pyruvate was shown to restore CD8^+^ TIL functions by increasing energy production by glycolysis and OXPHOS (112) (**Figure 1B4**). In an animal model of melanoma, pyruvate supplementation combined with checkpoint inhibitors targeting PD-1, CTLA-4, and T-cell immunoglobulin and mucin-domain containing-3 (TIM3) does not affect TIL already residing within tumors but increases enolase activity in newly infiltrating T cells allowing them to maintain functions and slow tumor progression (112).

Nevertheless, other pre-clinical studies have shown that some types of cancer cells can use pyruvate to fuel their metabolism (113). Clinical data are not yet available.

#### 2.6.2 Checkpoint inhibitors

A different type of drugs, referred to as biological drugs, which are remarkably effective in patients with unresectable or metastatic melanoma, are monoclonal antibodies against the checkpoint inhibitors PD-1 (pembrolizumab, nivolumab), PD-L1 (atezolizumab, durvalumab, avelumab) or cytotoxic T-lymphocyte associated protein 4 (CTLA-4, ipilimumab, tremelimumab) (114). These antibodies are thought to restore T cell functions by reversing T cell exhaustion. T cell exhaustion is irreversible at its later stages due to epigenic changes mediated by thymocyte selection-associated high mobility group box protein (TOX) that permanently silences genes producing key effector molecules while promoting expression of inhibitory markers (115). The effectiveness of checkpoint inhibitors is thus unlikely to solely reflect that they rescue TIL functions by reversing their exhaustion. As was shown in animals, checkpoint inhibitors modify the metabolic landscape of tumors by increasing glycolysis in TIL (12) and directly affecting tumor cell survival (62).

Checkpoint inhibitors have shown remarkable efficacy and in patients with treatment resistant melanoma nearly 50% achieve long-term control of their disease (116). Combining metabolic interventions with checkpoint blockade to further strengthen TIL responses is being investigated (117).

#### 2.6.3 Reprogramming the metabolism of TIL in situ

##### 2.6.3.1 PPARα agonists

Instead of changing the nutrient supply within the TME one can reprogram TIL to switch from glycolytic energy production to the use of OXPHOS fueled by fatty acids. The melanoma TME has elevated levels of fatty acids (62), which in part reflects that some tumors cause lipolysis of nearby adipocytes. This is mediated by tumor-derived cytokines and chemokines, which initiate a signaling cascade in adipocytes that increases levels of cyclic adenosine monophosphate (cAMP). The heightened activity of cAMP activates protein kinase A (PKA), which in turn phosphorylates and thereby stimulates hormone-sensitive lipase (HSL) that breaks down triglycerides into fatty acids (117). Other mechanisms that promote lipolysis in adipose tissues are the increased acidosis of tissues adjacent to tumors (118) or tumor cell-derived exosomes containing miR-425-3p. This miRNA promotes lipolysis by suppressing the activity of cAMP-specific 3’,5’-cyclic phosphodiesterase 4B; this increases cAMP levels, which in turn through PKA driven activation of lipases promotes lipolysis. The released fatty acids enter the TME where they can serve as nutrients for tumor cells as well as TIL.

**Figure 2:**
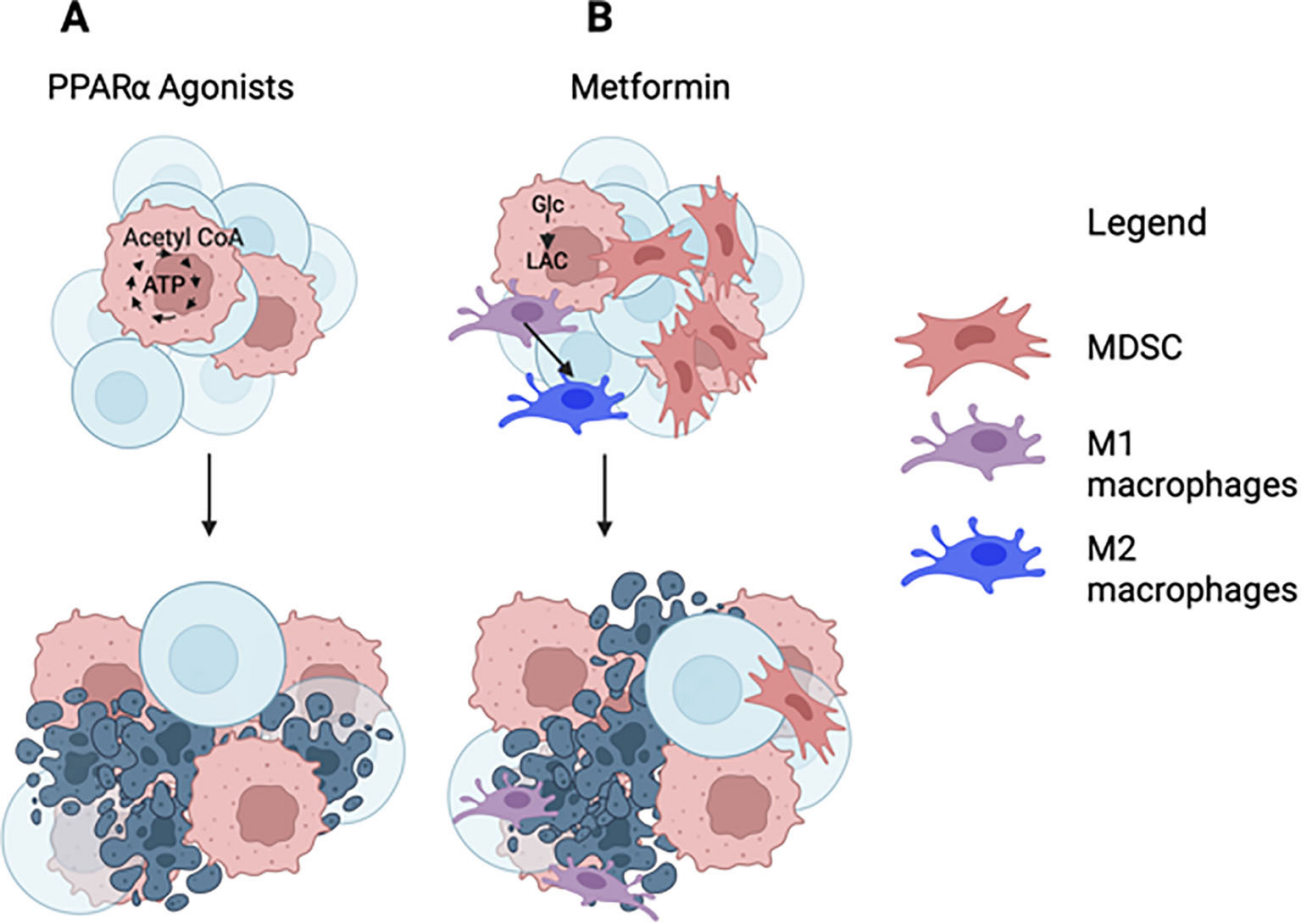
The effects of metabolic drugs on TIL. **(A)** PPARα agonists increase energy production through FAO and reduce glycolysis in TIL. **(B)** Metformin increases glycolysis in TIL; it also promotes macrophages to switch from an M2 to an M1 phenotype and reduces accumulations of MDSCs within the TME. **(A, B)** Both drugs improve T cell functions and viability. Created with BioRender.com.

TIL, when starving, switch to fatty acid metabolism as was shown by decreases in glycolysis metabolites accompanied by increases in TCA cycle metabolites (62). In addition, they enhance uptake of fatty acids and increase expression of Cpt1a. Reprogramming TIL from glycolysis to FAO can be reinforced by drugs such as fenofibrate that as a PPARα agonist promotes fatty acid catabolism while suppressing glycolysis (62) (**Figure 2A**). As we showed initially in a mouse melanoma model, fenofibrate treatment of TIL prior to their adoptive transfer into tumor bearing mice amplified their fatty acid uptake, expression of Cpt-1 and FAO resulting in increased expression of T-box expressed in T cells (T-bet). It also enhanced their functions and improved their ability to delay tumor progression. Similar results were obtained if fenofibrate was given directly to tumor bearing mice that had been vaccinated against a tumor-associated antigen (119). These results were further confirmed in a patient-derived xenograft (PDX) mouse melanoma model where ex vivo expanded CD8^+^ TIL were transferred into immunodeficient mice with homologous tumor transplants (120). Most strikingly were the effects of the PPARα agonist on TIL numbers within the TME. Tumors of mice that received ex vivo expanded CD8^+^ T cells that had not been treated with the drug showed only very low numbers of TIL within their TME when tested 4–6 weeks later as opposed to those transferred with drug pre-treated CD8^+^ T cells indicating that the drug-enforced metabolic switch either increased T cell survival or proliferation (**Figure 2A**).

Clinical data are not yet available.

##### 2.6.3.2 PPARδ agonists

Others have explored the use of PPARδ agonists for treatment of solid tumors. In one preclinical study the PPARδ agonist GW501516 was shown to increase FAO in activated CD8^+^ T cells, which increased their anti-tumor functions (121). In contrast, others found that in some types of cancers overexpression of PPARδ may increase tumor progression by enhancing angiogenesis and increasing tumor cell proliferation and survival (68).

##### 2.6.3.3 Metformin

Metformin, a drug commonly prescribed for type 2 diabetes, is an activator of AMPK, which induces cell cycle arrest and apoptosis in some types of cancer cells such as transformed melanocytes (122). It has additional effects on the TME and TIL. It is anti-inflammatory and switches differentiation of macrophages from the M2 towards the M1 phenotype (123). It reduces the accumulation of MDSCs within the TME through inhibition of production of the chemokine ligand 1 (CXCL1) chemokine by tumor cells (124). Through activation of AMPK leading to inhibition of the mevalonate pathway metformin reduces production of arginase in MDSCs thereby reducing their ability to suppress TIL functions (125).Through AMPK-mediated activation of PGC-1a it improves mitochondrial biogenesis and functions promoting OXPHOS and PPARα controlled metabolic pathways such as FAO in T cells (126) (**Figure 2B**). It increases numbers and functions of TIL by blocking their apoptosis and reducing T cell exhaustion (127). It was shown in preclinical studies to reduce O_2_ consumption by tumor cells and this in turn in combination with checkpoint blockade improved TIL functions and tumor clearance (128).

A meta-analysis of clinical trials with metformin used alone or in combination with other anti-cancer treatments showed marginal clinical benefits for cancers of the reproductive tract but indicated enhances progression of cancers of the digestive tract (129).

#### 2.6.4 *In vitro* manipulation of T cells

Enhancing the activity and longevity of T cells *in vitro* by shifting their metabolism has the clear advantage that it does not cause off-target side effects. Notwithstanding, unless metabolic reprogramming is achieved by genetic modifications, effects may be transient and wane once T cells are transferred into the patient and enter the TME.

##### 2.6.4.1 Reprogramming the metabolism of TIL *in vitro*

Many of the drugs that being given to experimental tumor-bearing animals or cancer patients to enhance TIL functions can also be used during ex vivo expansion of TIL or CAR-T cells. Examples are PPARα agonists (120), inhibitors of glycolysis, such as 2-deoxyglucose, which blocks the activity of hexokinase, the first enzyme of glycolysis (130), or inhibitors of the PI3K (e.g., alpelisib (131) and duvelisib (132)/AKT (e.g., ipatasertib and capivasertib (133) pathways. Alternatively, TIL can be forced to switch to OXPHOS by expanding them *in vitro* in a glucose-poor medium (134). This is technically challenging as lack of glucose reduces T cell proliferation and may therefore hinder the expansion needed to obtain clinically relevant quantities.

##### 2.6.4.2 Reprogramming the metabolism of CAR-T cells by bioengineering

**Figure 3:**
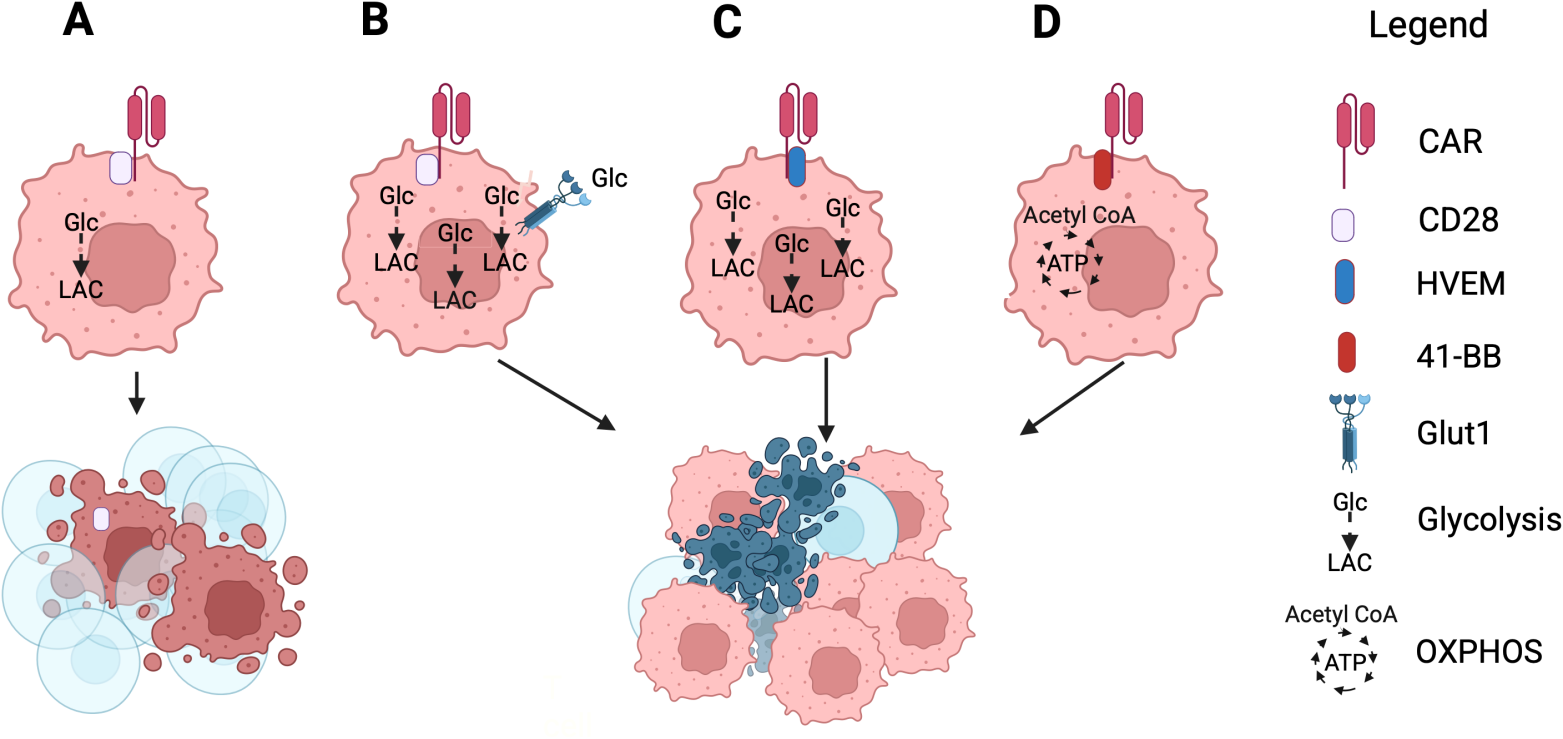
The effects of genetic modifications of CAR-T cells. **(A)** CAR-T cells commonly use the signaling domain of CD28 which drives their metabolism towards glycolysis resulting in their impairment within the TME. **(B)** CAR-T cells, which express increased numbers of Glut1 can outcompete tumor cells for glucose and thereby increase glycolysis and retain fitness. **(C)** CAR-T cells which use the signaling domain of HVEM increase glycolysis for energy production. **(D)** CAR-T cells which use the signaling domain of 4-1BB increase energy production through OXPHOS. **(B–D)** These metabolic changes improve CAR-T cell functions and viability. Created with BioRender.com.

CAR-T cells commonly use the intracellular CD3ζ domain together with the co-stimulatory domain of CD28, which skews their metabolism towards glycolysis (**Figure 3A**). Metabolism can be changed by generic modifications of CAR-T cells prior to their transfer. Glut1 overexpression allows for increased glucose consumption by CAR-T cells resulting in improved metabolic fitness and decreased differentiation towards exhaustion (135) (**Figure 3B**). Similarly using part of the herpes virus entry mediator (HVEM) instead of the CD28 domain as a co-stimulator was shown to improved functions of CAR-T cells by increasing glucose uptake, glycolysis, and lactic acid production (136) (**Figure 3C**). In an apparent contradiction to these results CAR-T cells with a costimulatory factor that favors OXPHOS such as 4-1BB (CD136), which unlike CD28 promotes AMPK signaling pathways, also showed improved survival within the TME (137) (**Figure 3D**), presumably reflecting that subtle differences within the TME dictate which pathway of costimulation is most appropriate to maintain CAR-T cell functions within the TME.

Superior CAR-T cell efficacy has been achieved by overexpression of an inhibition resistant, engineered version of PGC-1α (135). This resulted in metabolic reprogramming of the T cells which showed increased mitochondrial biogenesis, improved functions and superior control of tumor progression.

## 3 Summary

CD8^+^ T cell directed against tumor-associate antigens are the prime immune effector mechanism that can halt tumor progression. Nevertheless, they generally fail in part due to metabolic challenges TIL encounter within the TME. Upon activation CD8^+^ T cells switch to glycolytic energy production requiring glucose, which is in low supply within solid tumors. in addition, the TME is depleted of other key nutrients such as essential amino acids as well as O_2_. Activated CD8^+^ T cells can shift from glycolysis to alternative pathways of energy production. This adaptation seems to be inefficient and CD8^+^ T cells become exhausted and lose functions within the TME thus allowing for tumor progression, which aggravates the metabolic constrains within the TME. Medicinal interventions, which increase the supply of nutrients, especially glucose, in the TME or reprogram CD8^+^ T cells to use fatty acids for energy production have been shown to preserve their functions. Metabolic interventions may provide avenues to improve the outcome of active immunotherapy of solid tumors especially if combined with other immunotherapies such as checkpoint blockade.

Notwithstanding, the metabolism of tumor cells is extraordinarily heterogeneous; many rapidly growing tumor cells switch to glycolysis, others continue to use OXPHOS, or both. Tumor cell metabolism affects the nutrient supply within the TME, which in turn shapes the fate of TIL. This clearly provides challenges to metabolic interventions that aim to halt tumor progression by increasing the activity and longevity of TIL. One should thus not expect that metabolic intervention will evolve as one-fit all therapies but will rather once optimized become part of a personalized treatment regimen.
